# 2641. Nirsevimab binding-site conservation in RSV F protein between 2015 and 2022: The US OUTSMART-RSV surveillance study

**DOI:** 10.1093/ofid/ofad500.2253

**Published:** 2023-11-27

**Authors:** Christopher A Morehouse, Bahar Ahani, Anastasia A Aksyuk, Tyler Brady, Kevin M Tuffy, Hong Ji, Elizabeth J Kelly, Deidre Wilkins

**Affiliations:** AstraZeneca, Gaithersburg, Maryland; AstraZeneca, Gaithersburg, Maryland; Translational Medicine, Vaccines & Immune Therapies, BioPharmaceuticals R&D, AstraZeneca, Gaithersburg, MD, USA, Gaithersburg, MD; AstraZeneca, Gaithersburg, Maryland; AstraZeneca, Gaithersburg, Maryland; AstraZeneca, Gaithersburg, Maryland; AstraZeneca, Gaithersburg, Maryland; Translational Medicine, Vaccines & Immune Therapies, BioPharmaceuticals R&D, AstraZeneca, Gaithersburg, MD, USA, Gaithersburg, MD

## Abstract

**Background:**

Nirsevimab is an extended half-life monoclonal antibody that binds the prefusion conformation of RSV fusion (F) protein and has been approved for the prevention of respiratory syncytial virus (RSV) lower respiratory tract disease in neonates and infants in the EU, Great Britain, and Canada. Global surveillance studies found that the nirsevimab binding site was highly conserved across RSV variants between 1956–2021. While RSV B F protein binding site substitutions I206M and Q209R have dominated since the 2017/2018 RSV season, both remain fully susceptible to nirsevimab. OUTSMART-RSV is a multi-year (from 2015) prospective molecular surveillance study to monitor the prevalence and distribution of RSV variants in the USA and track the emergence and susceptibility of RSV F variants to nirsevimab.

**Figure:**
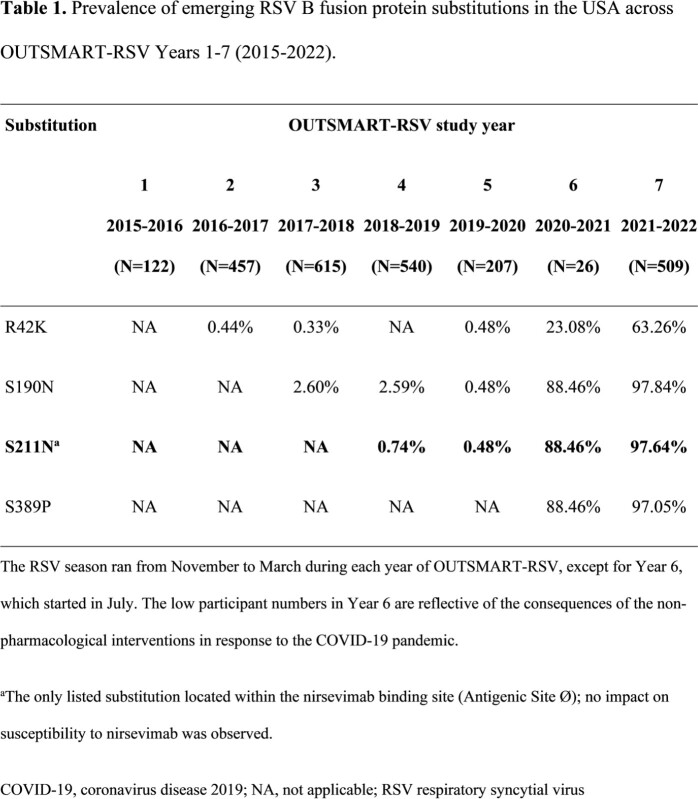

**Figure:**
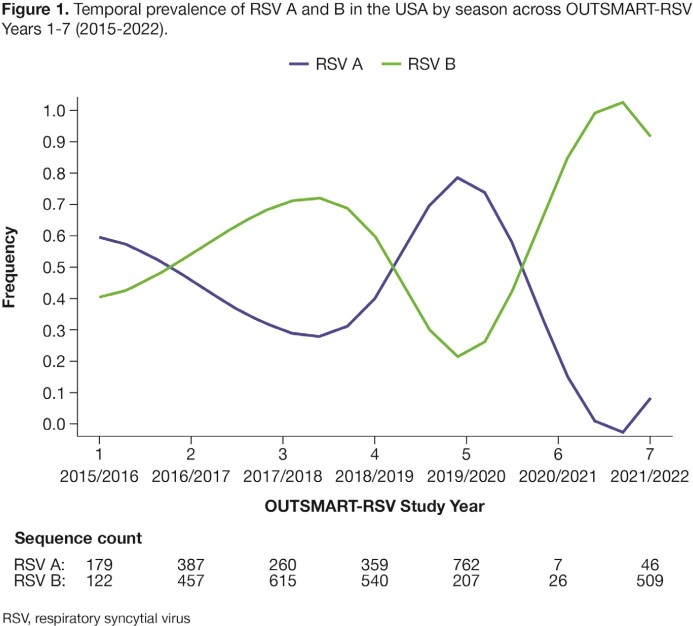

**Methods:**

Nasal swabs were collected from infants and adults seeking medical attention for respiratory infection (inpatient or outpatient); RSV-positive isolates underwent next generation sequencing. Subtyping comprised analysis of the hypervariable region of the G protein. F protein polymorphisms were identified by comparing with RSV A and B reference sequences; variants with increasing prevalence were phenotypically evaluated using reverse genetics rescue and *in vitro* microneutralization assays.

**Results:**

RSV A and B subtypes circulated with alternate frequency during OUTSMART-RSV (Figure 1), with RSV B dominant in the 2021/2022 RSV season (sequence count 509 vs 46 for RSV A; Table 1). From 2020, several additional RSV B F protein substitutions have increased in prevalence, including the S211N binding site substitution. Importantly, S211N retains full susceptibility to nirsevimab (IC_50_ fold change = 1.24 vs reference). Also, no new nirsevimab binding site substitutions were seen in the 2021/2022 RSV season vs previous seasons.

**Conclusion:**

While I206M and Q209R substitutions in RSV B F protein continue to dominate, the S211N nirsevimab binding site substitution has increased in prevalence since the 2020-2021 season. However, similar to I206M and Q209R, S211N variants remain fully susceptible to nirsevimab. Among recent circulating RSV strains, the frequency of emergent nirsevimab escape variants continue to be rare and not persistent.

**Disclosures:**

**Christopher A. Morehouse, MS**, AstraZeneca: Employee|AstraZeneca: Stocks/Bonds **Bahar Ahani, BSC**, AstraZeneca: Employee|AstraZeneca: Stocks/Bonds **Anastasia A. Aksyuk, PhD**, AstraZeneca: Employee|AstraZeneca: Stocks/Bonds **Tyler Brady, MS**, AstraZeneca: Employee|AstraZeneca: Stocks/Bonds **Kevin M. Tuffy, MS**, AstraZeneca: Employee|AstraZeneca: Stocks/Bonds **Hong Ji, MS**, AstraZeneca: Employee|AstraZeneca: Stocks/Bonds **Elizabeth J. Kelly, PhD**, AstraZeneca: Employee|AstraZeneca: Stocks/Bonds **Deidre Wilkins, BSC**, AstraZeneca: Employee|AstraZeneca: Stocks/Bonds

